# Mr. Oxley's Medical Essays

**Published:** 1801-04

**Authors:** Edward Oxley

**Affiliations:** Member of the Royal College of Surgeons of London, and Surgeon to the " Poor and Strangers Friend Society," at Hull. Hull


### *To the Editors of the Medical and Phyſical Journal.*

GENTLEMEN,

YOUR exertions in aid of MEDICAL Science, and, conſequently, in ſubſervience of the intereſts of ſuffering humanity, juſtly entitle you to the warmeſt thanks of the profeſſion, as well as to thoſe of the community at large.

At the commencement of your Journal, I was induced to believe it would eventually prove a highly uſeful and valuable medium of *medical* communication: the regular gradation of *intereſt* and *improvement* in the ſubſequent Numbers, hath amply juſtified the idea; and the plan you have adopted, renders it, in my opinion, more happily calculated for the diffuſion of *profeſſional* information, than any periodical publication which I am acquainted with in our language.

Under this conviction, and anxious to contribute my mite to the common fund, I have taken the liberty of tranſmitting you you a few FACTS, illuſtrative of the *hiſtory*, *nature*, and *medicinal properties* (chiefly) of certain *articles* and *preparations* of the *Materia Medica* and *Pharmacopœias*; which, from having long been conſidered as *dangerous* by ſome, and *inert* by others, had *nearly*, if not *wholly*, fallen into diſuſe.

What I have to ſtate of a *practical* nature, is the fair reſult of extenſive trial (for the moſt part) in the courſe of *my own* experience. For the ſake of method, I have arranged the ſeveral ſubjects in the *form* of *numerical*, and under the title of MEDICAL ESSAYS. Should the *facts* they contain be of the ſmalleſt *utility* to, and eſpecially were they to prove the *means* of exciting the *experimental reſearches* of other practitioners, it would afford me the moſt grateful ſatisfaction, from the conſideration that a well-directed enquiry into the *medicinal powers* of thoſe potent but imperfectly known phyſical treaſures, with which the ample kingdom of Nature is ſo abundantly ſtored, muſt neceſſarily be productive of the moſt *eſſential* and *permanent* advantage to MANKIND. I am, &c.

EDWARD OXLEY,

*Member of the Royal College of Surgeons of London, and Surgeon to the "Poor and Strangers Friend Society," at Hull.*

*Hull, November* 30, 1800.

---

### MEDICAL ESSAYS.

### No. I.

### *On the Hiſtory, Nature, and Medicinal Properties of the* ANTHEMIS PYRETHRUM, *Spaniſh Camomile, or Pellitory of Spain.*

SYNONYMA. Pyrethrum. *Pharm. Lond. et Edinb.* Pyrethrum flore bellidis, *Bauh. pin. p.* 14, 8. Pyrethrum officinarum. *Lob.* 447. *Gerarde Emac. p.* 758. *Park. Theat. p.* 858. *Raii. Hiſt. p.* 353. Chamœmelum ſpecioſo flore radice longa fervida. *Shaw Afric. p.* 138. Anthemis caulibus ſimplicibus unifloris decumbentibus. *Miller, fig. t.* 38. Πύρεθρον. *Dioſcorides, lib.* 3, *c.* 85.

*Claſs.* Syngeneſia. *Ord.* Polygamia ſuperflua, *Linn. Gen. Plant.* 970.

*Eſs. Gen. Ch. Recept.* paleaceum. *Pappus* nullus. *Cal.* hæmiſphæricus ſubæqualis. *Floſculi* radii plures quam 5.

*Sp. Ch.* A. caulibus ſimplicibus unifloris decumbentibus, foliis pinnato-multifidis. Vide *Woodville Med. Bot. vol.* 2. *p.* 286.

History, &c. The root is perennial, of the ſize of a man's finger, and of the colour of horſe radiſh, long, tapering, and ſendeth off numerous ſmall fibres. Stems, uſually ſimple, round, trailing, ſcarcely a foot in height, and bearing one flower; leaves, pinnate; segments, narrow, nearly linear, and of a pale green colour; flower, large at the diſc, of a yellow colour; at the radius, white on the upper, and of a purple colour on the under ſide: The different florets anſwer to the deſcription given of the anthemis *nobilis.* It flowereth in June and July.

The anthemis *pyrethrum* is a native of the Levant, and the warmer latitudes of Europe, but eaſily bears the winters of this climate; flowering from December until May, but doth not ripen its ſeeds here, unleſs the ſeaſon proves very warm and dry. It was firſt cultivated in England by Lobel, in 1570, and afterwards by Mr. Miller, in 1732: That obtained from abroad, however, is much the beſt for *medicinal purpoſes*; the only part employed for which, is the dried root; this comes to us in pieces of three or four inches in length, and from the ſize of a man's finger down to that of a common quill; (the ſmaller, as containing the greater quantity of reſinous matter, ſhould be preferred) when good, it is externally of a light brown colour, and hath a rough unequal ſurface; internally is of a whitiſh hue, and ſtriated texture; hath ſcarcely any perceptible ſmell, but if chewed in the mouth, diſcovereth a pungency of a very peculiar and powerful kind, exciting a copious flow of ſaliva; this ſtimulating quality is found to reſide in a fixed reſinous matter, of a deep red colour, which is lodged in numerous capillary receptacles, ſituate in the exterior, or cortical portion of the root. Rectified ſpirit extracteth the *whole* of its warmth; water very little; and no part of it is carried off in diſtillation or evaporation by either of theſe menſtrua.

Medicinal properties. It is an act of juſtice due to the memories of the antients, to obſerve of this truly valuable article, that, although neglected and almoſt unknown in *modern* practice, it hath been celebrated for its virtues from the dawnings of medical ſcience, and the far diſtant ages of antiquity. Dioscorides, the earlieſt author who wrote profeſſedly on the *Materia Medica*, and who is ſuppoſed to have lived not leſs than 1760 years ago, ſaith, "*it taketh away the cold ſhivering of agues, and is efficacious in the dead palſy.*" Gerarde, who wrote 200 years ago, ſpeaks of it as a powerful remedy in *chronic head-achs, vertigo, apoplexy, palſy, epilepſy, muſcular debility, and pains of the teeth, &c.* From the time of Gerarde to that of Cullen, the Engliſh authors of of repute whom I have had an opportunity of conſulting, make but very ſlight mention of the PYRETHRUM, and are, for the moſt part, *negative* as to its virtues, confining its uſe merely to that of a *maſticatory*. *Dr. Woodville*, however, in his elegant and intereſting work, the MEDICAL BOTANY, obſerves, with the greateſt propriety, that, "from the aromatic and ſtimulating qualities of the PYRETHRUM, there can be no doubt that it might be found an efficacious remedy, and equally fitted as an *internal* medicine, as many others of this claſs now conſtantly preſcribed." On this ground, of its *ſenſible qualities* alone, and ſeveral years ago, my firſt experiments were made; the favourable reſult of moſt of which, together with the ſucceſs of various ſubſequent trials, afford, in my opinion, the moſt decided confirmation of Dr. WOODVILLE's remark, as well as ample proof that DIOSCORIDES and GERADE, if fanciful in many inſtances, in this, at leaſt, ſpake the language of truth and experience.

It is peculiarly unfortunate for my preſent purpoſe, however, that owing to my not having ever entertained even the moſt diſtant idea of publiſhing, previous to the appearance of the MEDICAL JOURNAL, I committed ſuch *relative facts* as occurred in the courſe of my experiments with the pyrethrum, chiefly to *memory*; in conſequence of which, many important circumſtances have, through the lapſe of time, and other concurrent cauſes, eſcaped my recollection. The following (which, I truſt, will ſuffice to induce other practititioners to purſue the experimental enquiry) are amongſt the leading particulars which ſtrike my remembrance.

### CASES.

PARALYSIS *Rheumatica*. *Mary R——*, a poor woman, aged 46, of low ſtature, and extremely meagre and impoveriſhed habit, in conſequence of long expoſure to ſevere cold in the autumn of 1797, was ſeized with violent pain in the bowels and extremities, and two weeks after, completely loſt the uſe of the latter. I firſt ſaw this patient in the month of *November* following, ſome weeks after the attack of the complaint, and found her ſuffering the moſt excruciating torture, with ſcarcely an interval of eaſe; the whole frame emaciated and debilitated to the laſt degree; pulſe exceedingly feeble; appetite gone; and the upper and lower extremities much ſhrunk, contracted, and without the leaſt power of motion. Being a little inclined to coſtiveneſs, I preſcribed an aperient, namely, ℞. *Pil. aromat.* ʒiſs. *in pil. xviij. divid. ſum. iij. hora, ſomni. et repet. pro re nata.* Theſe procured a paſſage, and the ſucceeding morning gave the following: ℞. *Aq. menth. pip.* ℥7.

*ſpt. æther nitros. tinct. cinnam. c.* ℥vj. *m. fiat. miſt. ſum. coch.* iij. *mag. 4tis. horis.* This form was occaſionally varied; but no favourable change appearing, after a few days were elapſed, recourſe was had to full doſes of the higher claſs of *ſtimuli*, *Spt. æther vitr. æther vitriolic. tinct. opii. c. tinct. opii*, &c. were alternately adminiſtered, and the moſt powerfully ſtimulating embrocations were frequently applied to the extremities; theſe, however, affording only a very partial and temporary alleviation of the pain, and not the leaſt relief of the other ſymptoms, were laid aſide alſo, and the following ſubſtituted in their place. ℞. *Pulv. rad. pyrethri* ℈j. *Ol. ſaſſafras. gtt.* 5. *cons. cort. aurant. vel mucil. gum. Arab. q. s. fiat. bolus hora,* 11*ma. matutin. et hora ſomni ſumend.* A very ſenſible alteration for the better now took place, the patient experienced an almoſt *immediate* ceſſation of the pain, and in the courſe of a few days was able to waſh and dreſs herſelf: The doſe of the PYRETHRUM was then increaſed to *gr.* 50, and the *ol. ſaſſafras* to *gtt.* 10. *bis in die*; the reſult of which was, that in ſomewhat leſs than *two* weeks ſhe had ſo far recovered the uſe of her legs, as to be able to walk with the aſſiſtance of crutches; and by perſevering in the ſame plan for the ſpace of two weeks more, (excepting a ſlight contraction of her fingers) no ſymptoms of her malady remained; the pulſe was become natural, the torpor of the ſtomach was removed, the appetite returned, and ſhe was enabled to uſe her needle and attend her houſehold buſineſs as uſual.

PARALYSIS *Paraplegica. Elizabeth T——*, aged 47 years, of middle ſtature, and ſpare but not unhealthy habit, on the ceſſation of the catamenia, (which took place at 44), was attacked with almoſt inſupportably violent pain, alternately in the loins and region of the uterus, attended with conſiderable tumefaction of the abdomen. In this ſtate ſhe continued for the ſpace of *two years* and a half, during the latter part of which, her ſituation was rendered ſtill more peculiarly diſtreſſing by a ſupervening aſthmatic affection, the conſequence of imprudent expoſure to chill and damp air. Five months ago the pain *entirely* and *unexpectedly* left her, and eight weeks after this the abdominal tumour *wholly* and very *ſuddenly* ſubſided; the morning following which, on her attempting to raiſe from her bed, as uſual, ſhe found ſhe had completely loſt the uſe of her lower extremities, which were cold, rigid, and perfectly inſenſible to the touch. I was firſt requeſted to attend her the 26th of *October* laſt; found her ſituation as above deſcribed, with the pulſe natural, the evacuations healthy and regular, and the appetite good. Gave the following bolus: ℞. *Pulv. rad. pyrethri.* ʒſs. *syr. cort. aurant. q. s. fiat. bolus hora ſomni ſumend*; and to to allay the ſpaſmodic irritation in the cheſt, ℞. *Aq. menth. pip.* ℥j. *spt. æther. nitro. tinct. opii. camph. aa.* ℥ſs.——*Zinzib.* ʒiij. *m. ft. miſt. sum. coch. iij. mag. 4tis. horis.* This enabled the patient to breathe more freely, allayed the extreme violence of the cough, and procured a few hours refreſhing reſt, which ſhe had long been prevented the enjoyment of. *October 27th and 28th*, the *boluses* were repeated; on the 29th, the warmth and ſenſibility of the lower extremities returned, and the rigidity was ſo far overcome as to admit of the free and alternate motion of the joints. In compliance with the wiſhes of the patient, the Pyrethrum was omitted for five days, at the expiration of which, namely, on the 2d of the preſent month *November*, it was re-employed in the following (as a leſs ungrateful) form, ℞. *Pulv. rad. pyrethri.* ʒj. *ol. menth. pip. gtt.* 5. *cons. cort. aurant. vel mucil. gum. Arab. q. s. fiat. pil. No.* 18. *ſumat iij. bis in die:* And to obviate coſtiveneſs, gave occaſional and mild aperients. *November* the 5th, the patient could walk about the room with the aſſiſtance of chairs, but at this period experienced a very ſevere attack of *cholera*, (the prevailing epidemic) which reduced her ſtrength conſiderably, and the Pyrethrum was once more neceſſarily omitted for eighteen days, in order to make way for other remedies. November 24th, again had recourſe to it, as follows: ℞. *Pulv. rad. pyrethri.* ʒiſs. *mel. anglic. q. s. fiat. pil. No.* 36. *sumat. iij. ter in die.* Under this form, have continued its exhibition until the preſent time, and with evident and daily-increaſing advantage to the patient, who hath now regained ſufficient ſtrength to enable her to walk with very ſlight ſupport; and if it is allowable to infer from previous and ſucceſsful experience, have no doubt that in a few weeks I ſhall have it in my power to announce her entire recovery.

Lumbago. *Thomas S*——, ſeaman, aged 26 years, of tall ſtature, ſtrong, athletic make, and remarkably healthy habit, on the 10th of *November* inſtant, was ſeized with very ſevere pain and great rigidity in the muſcles ſituate on the loins; on the 12th I was requeſted by the owner of the ſhip to which he belonged, to attend him, and ordered the following: ℞. *Spt. terebinth. ol. olivar. aa.* ʒvj. *aq. ammon. pur.* ℥ſs. *M. fiat. liniment. purt. affect. ter in die applicand.* ℞. *Tinct. opii. gtt.* 60. *T. opii, camph. ſpt. æther. nitros. aa.* ℥ſs. *M. sumat. coch. j. parv. 4tis. horis, in aq. font.* On the 13th found the ſymptoms more violent than before, and the patient complaining of coſtiveneſs, gave as follows: ℞. *Calomel. gr. vj. cons. cort. aurant. q. s. fiat. bolus hora somni ſumend:* ℞. *Infus. sennæ* ℥vjſs. *natron. vitriol.* ℥iſs. *ſacchar. rubr.* ℥ſs. *M. fiat. mist. sum. coch. iij. mane. et repet omni hora pro re nata.* Theſe anſwered the purpoſe pose of opening the bowels, but yielded no other relief. On the 16th, the pain and stiffness were precisely the same, and the integuments of the part had acquired a livid hue. The form of the anodyne, prescribed in the first instance, was now varied, and re-employed as follows: ℞. *Tinct. balsam Peru* ℥j. *Opii gtt. lx. fiat. tinct. sum. coch. j. parv. 4ts. horis. in aq. hordeat.* This afforded a momentary alleviation of the pain, which however returned on the 17th, as severe as before. Baffled in my expectations from every means hitherto employed, had recourse to my *forlorn hope*, and favourite remedy, the PYRETHRUM, in the following form: ℞. *Pulv. rad. pyrethri.* ʒij. *mel. anglican. q. s. fiat. elect. sum. coch. j. parv. 3tiis. horis.* The first dose abated the violence of the symptoms; in about 18 hours, the patient had taken the whole of the electuary, and in little more than 24, the pain and stiffness were entirely gone, the integuments had re-gained their natural colour, and not a trace of the malady remained.

INTERMITTENTES. *Pyrethrum* furnishes a highly useful addition to the *cinchona*, in the cure of intermittents; I have employed it in conjunction with the latter article, in various cases of obstinate TERTIAN, and with uniformly good effect. On those occasions, it was commonly exhibited under the following, as the most eligible form. ℞. *Pulv. rad. pyrethri.* ʒij. *pulv. cort. cinchonæ, flav.* ℥j. *syr. cort. aurant. q. s. fiat. elect. sum. coch. j. parv. 3tiis. horis absente febre.* The mention of the efficacy of the PYRETHRUM, in cases of this nature, recalls to mind a rather curious circumstance related to me some time ago. An ingenious mechanic, formerly a neighbour of mine, having read in some old author of the virtues of the pellitory of Spain, undertook to administer it in large doses to persons labouring under ague, and found it, in all respects, a most effectual specific. His invariable plan of treatment was, immediately on the accession of the chill stage, to order the patient to bed, then give two drachms of the powdered root in warm ale, one drachm at the end of two, and the same quantity at the end of six hours, in like manner; a profuse sweat ensued, and on its going off, the patient rose perfectly recovered. And further, however improbable or remarkable it may appear, out of *hundreds* in which this *red-hot* practice was adopted, not a *single instance* occurred, in which it failed of success. The intimate personal knowledge I had of the character of the man, leaves me no room to doubt the truth of the relation, which I had from his own mouth.

ODONTALGIA. *Pyrethrum* hath long been in high request with the *dentists*, and forms the basis of various advertised nostrums for the *tooth-ache*: its efficacy, indeed, in cases of this kind, kind, hath from time immemorial been known to the vulgar, who frequently find a remedy in the ſimple maſtication of the root. In thoſe *rheumatic affections* of the *maxillæ*, to which many perſons are ſubject in certain ſtates of the atmoſphere, I have occaſionally ſeen it peculiarly uſeful, either employed in the manner above-mentioned, or in the form of pills or tincture, as follows: ℞. *Pulv. rad. pyrethri.* ℈j. *mucil. gum. arabic. q. s. fiat. pil.* 12. ℞. *Pulv. rad. pyrethri.* ℥x. *spt. æther. vitriol. C. vel spt. vinos. rect,* ℔j. *infund per dies decem, et cola, postea adde camphor.* ℥j. *ol. roresmarin.* ℥ſs. *tinct. opii* ʒij. *fiat. tinct.* When the tenderneſs or tumour of the gums had rendered the maſtication of the root impracticable, one or more of the pills (firſt dried, in order to render their ſolution more gradual,) were held in the mouth, until diſſolved, or a piece of lint moiſtened with the tincture, was applied to the painful part, and repeated as occaſion required.

Paraphonia *rauca.* In ſeveral caſes of *chronic hoarſeneſs* and *deprivation of the voice*, ariſing from ſevere attacks of catarrh, and attended with tumour and relaxation of the fauces, as a topical application, I have made uſe of the following watery infuſion of the pyrethrum. ℞. *Pulv. rad. pyrethri.* ʒij. *ad.* ℥ſs. *infund. in aq. bullient.* ℥8. *per horas duas cola et adde spt. vinos. rect.* ℥ſs. *fiat. gargarism.* frequenter utend. (A form ſomewhat ſimilar is employed in St. Bartholomew's Hoſpital.) In ſome few inſtances, this was productive of at leaſt temporary benefit to the reſpective patients; but as the *pellitory* giveth out its active principle very imperfectly to aqueous menſtrua, permanent advantage can ſcarcely be expected from *formulæ* of the kind above-mentioned; I have no doubt, however, that a uſeful remedy would be found in a ſimple tincture of the root, on the like occaſions.

In addition to the foregoing, numerous other inſtances of the efficacy of the anthemis *pyrethrum* (which have occurred ſince the commencement of this eſſay,) might be ſtated, but the facts adduced above may probably ſuffice to promote a more extenſive trial of its virtues. Before cloſing the ſubject, however, a few practical obſervations on the *doſe*, *form of exhibition*, *mode of operation*, and *ſenſible effects* of this remedy, may not be altogether improper.

In ordinary practice, the doſe is from ten to forty grains, every four, five, or ſix hours, according to the age and ſtrength of the patient, and the circumſtances of the caſe: but when an extraordinary ſtimulus is required, experience hath ſhewn, that it may be extended to ℥ſs, or even more, in the courſe of the 24 hours; and with the beſt effects, provided it be given in in fuch portions, and at fuch intervals as the ftomach can conveniently bear.

Thefe remarks will, of courfe, be underftood, as referring to the *pyrethrum* in fubftance, in which ftate only, and from neceffity rather than choice, I have hitherto employed it for internal purpofes, for the moft part combined with *honey*, or *mucilage*, in the form of *bolus* or *electuary*. The quantity, however, requifite on many occafions for a dofe, added to its unpleafant pungency, renders it to moft, a naufeous and ungrateful medicine. It is much to be wifhed, therefore, that the fhops could be fupplied with an *extract*, carefully prepared with rectified fpirit, which, as poffeffing the virtues of the root, in a fmall volume, and concentrate ftate, would be abundantly preferable to every other form.

As it is intended that the "THEORY OF STIMULI" fhould form the fubject of a future effay, in elucidation of the *modus operandi*, it may fuffice for the prefent, to remark in a brief and general way: *Firft*, That in *local* affections connected with, or dependent on *difeafed action* of, or *deficiency of power* in fome part of the *mufcular* or *nervous fyftems*, it appears, with certain conftitutions, to operate as a *fympathetic* or *indirect ftimulus*, moft probably by exciting in the nervous diftribution of the ftomach, (the *main fpring* of our mechanifm,) a *fpecific* and *healthy action*, which through the fame medium of the nerves, *fympathetically* counteracts the morbid and irregular motions, or overcomes the torpor and debility conftituting the complaint.

*Secondly*, In other habits, and in fimilar affections, it acts as an *immediate* or *direct ftimulus*, producing exactly analogous effects, by a *local* application of its active principle (conveyed through the channel of the circulation) to the part affected.

The firft of thefe *rationalia*, feems fupported by the fometimes *fudden*; and the fecond, by the more generally *gradual* manner, in which the *pyrethrum* allays rheumatic pains of the joints and extremities, removes the rigidity, and reftores the loft warmth of paralytic limbs.

In this *two-fold mode of operation* then, namely, as a *fpecific ftimulus* and *local tonic*, does this ufeful remedy (when judicioufly managed) tend, in different difeafes deriving their origin from indirect debility, to reftore and maintain the EQUILIBRIUM OF HEALTH; exhibited in an over-dofe, however, or without that previous preparation which peculiarity of conftitution or accidental circumftances frequently renders requifite, it occafionally excites the moft inordinate and violent action in various parts of the fyftem, fuch as naufea of the ftomach and fevere vomiting, tormina of the bowels, and exceſſive diarrhœa, diſtreſſing head-aches, and painful anxiety, eſpecially in bilious habits and ſuch as labour under habitual dyſpepſia. In the firſt inſtance, therefore, it will be neceſſary to premiſe an *emetic*; in the latter, ſome mild *ſtomachic*; and in every caſe, coſtiveneſs muſt be obviated by occaſional *aperients*, ſuch as the *Pulv. rhabarbari*, *pil. ex aloe cum myrrh or ol. ricini.*

Laſtly, Should the length to which this ESSAY has been extended appear to require an apology, the writer would beg leave to remark, that from a wiſh to reſcue from neglect and oblivion, one of the moſt *potent* and *valuable* articles of the MATERIA MEDICA, he was induced to prefix to the various proofs of its *medicinal* powers, with which individual experience and attentive obſervation had furniſhed him, the *botanical*, *natural*, and *medicinal* hiſtory of the ANTHEMIS *pyrethrum*, under the conviction of its contributing to that end; and conceives ſuch addition will not be conſidered ſuperfluous or impertinent, when (as containing beſides ſome original, he believes every ſcattered ray of relative information collected into one focus from the moſt reſpectable authorities,) it not only ſupplies a comprehenſive and ready reference, (particularly to thoſe not in poſſeſſion of the valuable works of *Gerarde* or *Dr. Woodville)* but may alſo in a great meaſure ſerve as a *neceſſary guide* to ſuch practitioners as are not previouſly acquainted with the *pyrethrum*, in their choice of it, (on which, in the firſt inſtance, all hopes of ſucceſs depend) as what is found in the ſhops is commonly ſo much injured by time, damp, or worms, as to be utterly unfit for uſe.

In reference to the reſpective CASES, it is hoped, that as the ſeveral circumſtances of each are detailed with the ſtricteſt fidelity, although tedious in the relation, they will be found poſſeſſing ſufficient importance to plead their own apology.

It is with ſome a matter of regret, however, that the *literary labours* of thoſe engaged in the *medical* profeſſion do not always poſſeſs the advantages of *correctneſs* of compoſition and *elegance* of ſtile. Deeply aware of the numerous defects of that nature in the preſent attempt, the Author can only obſerve, that while the momentous and indiſpenſible engagements of profeſſional men allow them little leiſure to attend to the *extrinſic embelliſhments* of language, they afford ample opportunity for the attainment of what is of infinitely greater conſequence, (and of which he truſts that candour will allow he has availed himſelf to the utmoſt) *intereſt* and *accuracy* in point of FACT.

